# The Mediating Role of Disgust Sensitivity in the Relationship Between Professional Perception and Professional Belonging Among Midwifery Students: A Cross‐Sectional Study

**DOI:** 10.1002/nop2.70749

**Published:** 2026-08-16

**Authors:** Betül Kaplan, Songül Kekil, Ayşe Elkoca

**Affiliations:** ^1^ Midwifery Department, Faculty of Health Sciences Gaziantep Islam Science and Technology University Gaziantep Türkiye

**Keywords:** disgust sensitivity, midwifery education, midwifery students, professional belonging, professional identity

## Abstract

**Aim:**

To examine the relationships among disgust sensitivity, professional perception and professional belonging in midwifery students, and to test whether disgust sensitivity statistically mediates the perception–belonging association.

**Design:**

A descriptive, cross‐sectional, correlational study.

**Methods:**

Between March and May 2025, 295 midwifery students at a state university in southeastern Türkiye completed an online survey comprising a sociodemographic form and three validated scales measuring disgust propensity and sensitivity, midwifery vocational perception and midwifery belonging. Data were analysed with descriptive statistics, *t*‐tests, analysis of variance, correlation, hierarchical regression and bootstrap mediation analysis.

**Results:**

Students reported high levels of professional perception and belonging and a moderate level of disgust sensitivity (interpreted relative to each scale's possible range). Professional perception was positively associated with belonging, while disgust sensitivity was negatively associated with both. Hierarchical regression showed that professional perception was the strongest predictor of belonging, with disgust sensitivity making a small additional negative contribution (4.2% of variance). Bootstrap analysis indicated that disgust sensitivity partially and statistically mediated the perception–belonging association.

**Conclusion:**

Professional perception is the strongest correlate of professional belonging in midwifery students, and disgust sensitivity shows a small negative association that partially and statistically accounts for the perception–belonging relationship. Because the design is cross‐sectional, these associations should not be interpreted causally.

**Implications for the Profession and/or Patient Care:**

Nurse and midwife educators and clinical mentors can use these findings to support students who experience disgust during placements. Practical, evidence‐based applications include normalising disgust in clinical supervision, embedding graduated and simulation‐based exposure before high‐contact procedures, providing structured reflective debriefing after emotionally demanding events such as perinatal loss, and offering supportive mentorship—measures that may help preserve caring behaviour and strengthen students' connection to the profession.

**Impact:**

The study addressed whether disgust sensitivity is associated with professional belonging in midwifery students and how it relates to the perception–belonging link. Findings show that professional perception is the strongest correlate of belonging and that disgust sensitivity is independently and negatively associated with belonging and partially mediates this relationship statistically. The findings inform nurse and midwife educators, curriculum designers and clinical mentors, particularly regarding clinical placement and emotion‐focused educational support.

**Reporting Method:**

STROBE checklist for cross‐sectional studies.

**Patient or Public Contribution:**

No patient or public contribution. Midwifery students were the participants but were not involved in the design, conduct or reporting of this study.

## 1 Introduction

Midwives play a central role in the protection and promotion of maternal and neonatal health and are a key workforce in achieving the Sustainable Development Goals related to reproductive, maternal, newborn and adolescent health (Adnani et al. 2025; Khan and Yildirim 2024). The scope of contemporary midwifery practice ranges from preconception counselling, antenatal monitoring and physiological birth management to early identification and referral of complications, immediate newborn care, breastfeeding support and family planning (Combellick et al. 2023). Education aligned with international competency standards is therefore a prerequisite for safe, evidence‐based and woman‐centred midwifery care.

The quality and safety of midwifery care depend not only on technical competence but also on whether students develop a durable professional identity and remain in the profession. Sense of belonging and positive professional perception are established correlates of professional commitment, retention and wellbeing in nursing and midwifery, and are therefore directly relevant to workforce sustainability and, ultimately, to the quality of woman‐centred care (Mbalinda et al. 2024; Yıldırım et al. 2022).

Although disgust is a routine emotional experience during clinical placement, it remains an under‐discussed and rarely addressed topic in nursing and midwifery education (Cho et al. 2023; Hadjittofi et al. 2022). Existing nursing research has largely documented that students experience disgust and that higher disgust sensitivity may be associated with reduced caring behaviours (Doğan et al. 2025; Özkan et al. 2021), but it has stopped short of linking disgust to the professional concepts—perception and belonging—that underpin recruitment, retention and the quality of nursing and midwifery care. This is the specific nursing knowledge gap that the present study addresses. The unmet need is practical: nurse and midwife educators currently lack evidence on whether, and how strongly, students' emotional responses relate to their sense of belonging, evidence that is required to justify and design emotion‐focused educational support within nursing and midwifery curricula. By quantifying these associations and testing disgust sensitivity as an intervening variable, this study advances nursing knowledge from the observation that disgust exists towards an evidence base that can inform how nursing and midwifery programmes prepare students for the affective demands of clinical practice.

## 2 Background

Clinical placement is a defining feature of midwifery education and the principal arena in which students develop technical competence, professional values and a sense of belonging to the profession (Folkvord and Risa 2023; Sun et al. 2022). Professional belonging is conceptualised as the identification of an individual with a profession and the establishment of an emotional connection with its values, roles and community of practice; a strong sense of belonging has been linked to professional commitment, retention, motivation and well‐being among nursing and midwifery students (Mbalinda et al. 2024; Yıldırım et al. 2022). Closely related is professional perception, which captures students' cognitive and affective evaluations of their profession—their understanding of its roles, professionalism and responsibilities (Bilgin and Merih 2021). Positive professional perception has been associated with the development of professional identity, increased satisfaction and motivation, and better quality of care (Arundell et al. 2024; Demir and Taşpınar 2021).

Alongside these cognitive and identity‐related processes, midwifery students are routinely exposed to body fluids, secretions, intimate examinations and procedures with infection risk, and to morally and culturally challenging situations such as perinatal loss. These exposures can elicit disgust, an emotion conceptualised within the Behavioural Immune System framework as an evolved psychological mechanism that protects individuals from pathogen contact by motivating avoidance of contaminating cues (Troisi et al. 2022; Van Overveld et al. 2006). Disgust sensitivity refers to the degree of distress an individual experiences in response to disgust‐eliciting stimuli, while disgust propensity reflects the frequency with which the emotion is triggered (Arusoğlu 2021). These are related but distinct constructs, and researchers caution that findings obtained with one should not be assumed to transfer directly to the other; the present study therefore focuses on disgust sensitivity while measuring both with the same validated instrument (Van Overveld et al. 2006). Although the Behavioural Immune System provides an adaptive defence against infection, it can also generate avoidance behaviours that conflict with the caring expectations placed upon healthcare professionals (Hadjittofi et al. 2020, 2022; Salmani Mood and Nasiri 2025).

A growing body of evidence indicates that disgust is a common but under‐discussed experience among nursing and midwifery students and is associated with a range of clinically relevant outcomes. Phenomenological studies have shown that students often struggle to talk about disgust and use coping strategies such as relabelling, professional distancing and reliance on empathy (Cho et al. 2023; Hadjittofi et al. 2022). Quantitative studies have linked higher disgust sensitivity with reduced caring behaviours among nursing students and clinical nurses (Adagide et al. 2024; Doğan et al. 2025; Özkan et al. 2021; Song et al. 2024) and with lower willingness to care for older adults (Adagide et al. 2024). Qualitative work in medical and midwifery education further suggests that intense disgust can disrupt empathic engagement and threaten professional identity construction (Warmington et al. 2022). Despite this evidence, no study to our knowledge has examined whether disgust sensitivity is associated with the link between professional perception and professional belonging.

There is a theoretical basis for expecting disgust sensitivity to lie on the pathway between professional perception and professional belonging. Appraisal‐based accounts of the Behavioural Immune System hold that the intensity of disgust depends partly on how a stimulus is cognitively appraised: students who perceive midwifery as meaningful and valuable may reframe potentially aversive exposures (e.g., body fluids, intimate examinations) as purposeful aspects of care, which could attenuate the disgust they experience (Troisi et al. 2022). Lower experienced disgust, in turn, may reduce avoidance impulses that conflict with caring norms and thereby support a stronger sense of fit with—and belonging to—the profession (Hadjittofi et al. 2020). On this reasoning, disgust sensitivity is proposed as a candidate intervening (mediating) variable linking professional perception to professional belonging. Because all variables in the present study were measured concurrently, this pathway is examined as a statistical association rather than a causal sequence.

## 3 The Study

### 3.1 Aim(s) and Objective

The aim of this study was to examine the levels of disgust sensitivity, professional perception and professional belonging in midwifery students, to explore the relationships between these variables and selected sociodemographic and clinical characteristics, and to test whether disgust sensitivity statistically mediates the association between professional perception and professional belonging. The comparisons across sociodemographic and professional characteristics (research question 2) were planned as exploratory analyses, intended to describe the sample and generate hypotheses rather than to test pre‐specified directional predictions. The following research questions guided the study:

What are the levels of disgust sensitivity, professional perception and professional belonging among midwifery students?

Do disgust sensitivity, professional perception and professional belonging differ by sociodemographic and professional characteristics (e.g., year of study, professional satisfaction, perceived professional identity)?

Is there a significant relationship between professional perception, professional belonging and disgust sensitivity in midwifery students?

Does disgust sensitivity statistically mediate the relationship between professional perception and professional belonging?

## 4 Methods

### 4.1 Design

This study was designed as a descriptive, cross‐sectional and correlational study and reported in accordance with the Strengthening the Reporting of Observational Studies in Epidemiology (STROBE) guidelines for cross‐sectional studies (see File S1).

### 4.2 Setting and Participants

The research took place in the Department of Midwifery, Faculty of Health Sciences, of a state university located in southeastern Türkiye [institution name withheld for blinded peer review]. Eligible participants were students who (i) were officially enrolled in the midwifery programme in the 2024–2025 academic year; (ii) were at least 18 years of age; and (iii) volunteered to participate. Students who did not give informed consent or who did not completely fill in the questionnaire were excluded.

### 4.3 Sample Size and Power

The accessible population consisted of 352 students. The minimum required sample size was calculated using Cochran's formula at a 95% confidence level and a 5% margin of error, yielding 184 participants. To enhance representativeness and to compensate for potential dropouts, a target of approximately 85% of the population was set; the final sample comprised 295 midwifery students (response rate 83.8%).

### 4.4 Data Collection

Data were collected between March and May 2025 using a self‐administered online questionnaire delivered through Google Forms. The link was distributed via official student communication channels with the support of class representatives. Participation was voluntary, and informed consent was obtained on the first page of the survey before any items were displayed. The questionnaire took approximately 10–15 min to complete.

### 4.5 Instruments

#### 4.5.1 Personal Information Form

A 10‐item form developed by the researchers based on the relevant literature was used to collect sociodemographic and professional characteristics, including age, year of study, reason for choosing the profession, professional satisfaction, career preference, perceived professional identity, perception of the future of the profession, experience of disgust during clinical practice, presence of negative thoughts about the profession, avoidance of care and self‐doubt regarding professional competence (Yıldırım et al. 2022; Yılmaz and Siyahtaş 2024).

#### 4.5.2 Disgust Propensity and Sensitivity Scale–Revised (DPSS‐R)

The Disgust Propensity and Sensitivity Scale–Revised (DPSS‐R) was developed by Van Overveld et al. (2006), and its Turkish validity and reliability were established by Arusoğlu (2021). It contains 16 items rated on a 5‐point Likert scale (1 = never, 5 = always) and yields two subscale scores—disgust propensity (8 items) and disgust sensitivity (8 items)—and a total score, with higher scores indicating greater disgust propensity and sensitivity. All items are positively scored. The Cronbach's alpha reported for the Turkish version was 0.91 (Arusoğlu 2021); in the present study, Cronbach's alpha was 0.91 for the total scale, 0.85 for disgust propensity and 0.83 for disgust sensitivity.

#### 4.5.3 Midwifery Vocational Perception Scale (MVPS)

The Midwifery Vocational Perception Scale (MVPS) was developed by Bilgin and Merih (2021) and contains 16 items rated on a 5‐point Likert scale (1 = strongly disagree, 5 = strongly agree). It comprises three subscales—Role Perception (items 1–6), Perception of Professionalism (items 7–12) and Perception of Duties and Responsibilities (items 13–16). Total scores range from 16 to 80, with higher scores indicating a more positive perception of midwifery. The Cronbach's alpha for the original scale was 0.86 (Bilgin and Merih 2021); in the present study, Cronbach's alpha was 0.96 for the total scale and ranged from 0.86 to 0.92 across the three subscales.

#### 4.5.4 Midwifery Belonging Scale (MBS)

The Midwifery Belonging Scale (MBS) was developed by Başkaya et al. (2020) and consists of 22 items rated on a 5‐point Likert scale (1 = strongly disagree, 5 = strongly agree), with no reverse‐coded items. Total scores range from 22 to 110, with higher scores indicating a stronger sense of belonging to the midwifery profession. The scale comprises four subdimensions: emotional belonging, fulfilling professional roles and responsibilities, evaluating professional development and opportunities, and limits of duties and authority in the profession. The Cronbach's alpha reported for the original scale was 0.90 (Başkaya et al. 2020); in the present study, Cronbach's alpha was 0.93 for the total scale and ranged from 0.71 to 0.90 across the four subscales.

### 4.6 Data Analysis

Data were analysed using IBM SPSS Statistics, version 30 (IBM Corp., Armonk, NY, USA). Continuous variables are presented as mean ± standard deviation (SD) and categorical variables as frequency and percentage. The normality of continuous variables was assessed using the Kolmogorov–Smirnov test, skewness and kurtosis values (within ±1.5), histograms and Q–Q plots. Because the Kolmogorov–Smirnov test tends to be sensitive to departures from normality in large samples, primary reliance was placed on the visual indicators and skewness/kurtosis values, in line with Pallant (2020). Variables met the assumptions for parametric testing.

For descriptive interpretation, mean scale scores were expressed as a percentage of the maximum attainable score and described as low (< 50%), moderate (50%–75%) or high (> 75%). These verbal descriptors are interpretive aids relative to each instrument's possible range and do not represent validated clinical cut‐offs.

Bivariate group comparisons were conducted only for variables in which every subgroup had an adequate cell size. Independent‐samples *t*‐tests were used to compare two independent groups (perceived professional identity, experience of disgust, negative thoughts, avoidance of care, doubt about competence). One‐way analysis of variance (ANOVA) was used for comparisons among more than two adequately sized groups (year of study, professional satisfaction), and Welch's test was applied when Levene's test indicated a violation of the homogeneity of variances assumption. Where ANOVA or Welch tests were significant, Bonferroni‐corrected or Games–Howell post hoc tests were used as appropriate, and significant pairwise differences are reported in the text and in Table 3. Categorical variables containing small subgroups (career preference, reason for choosing the profession and perception of the future of the profession) were not entered into inferential group comparisons and are reported only descriptively in Table 1. Pearson correlation analysis examined relationships among the total scale scores. Given the number of comparisons, particular emphasis is placed on associations significant at *p* < 0.001, which would survive a Holm–Bonferroni adjustment.

**TABLE 1:** Sociodemographic and professional characteristics of the midwifery students (*N* = 295).

Variable	Mean ± SD/*n*	Min–Max/%
Age (years)	20.68 ± 2.55	18–38
Year of study		
1st year	81	27.5
2nd year	96	32.5
3rd year	62	21.0
4th year	56	19.0
Reason for choosing the profession		
Conscious and research‐based preference	66	22.4
Family‐based guidance and preference	40	13.6
Recommendation of close environment/friends	14	4.7
Job opportunities and career prospects	116	39.3
Choice based on academic achievement/score	59	20.0
Professional satisfaction		
Yes	160	54.2
No	30	10.2
Undecided	105	35.6
Career preference		
Academia	39	13.2
Independent midwifery practice	38	12.9
Public hospitals	191	64.7
Private hospital	7	2.4
Non‐midwifery field	9	3.1
Other	11	3.7
Perceived professional identity		
Yes	267	90.5
No	28	9.5
Perception of the future of the profession		
Optimistic	188	63.7
Pessimistic	11	3.7
Neither optimistic nor pessimistic	96	32.5
Experience of disgust during clinical practice		
Yes	90	30.5
No	205	69.5
Negative thoughts about the profession		
Yes	81	27.5
No	214	72.5
Avoidance of care		
Yes	28	9.5
No	267	90.5
Doubt about professional competence		
Yes	38	12.9
No	257	87.1

*Note:* Career preference, reason for choosing the profession and perception of the future of the profession are reported here as descriptive characteristics only and were not used in inferential group comparisons because of small subgroup sizes.

Abbreviations: %, percentage; Max, maximum; Min, minimum; *n*, frequency; SD, standard deviation.

Hierarchical regression analysis was used to examine the predictive contribution of professional perception and disgust sensitivity (total scores) to professional belonging, with multicollinearity assessed via tolerance (> 0.2) and variance inflation factor (VIF < 10) values and independence of residuals via the Durbin–Watson statistic. Mediation was tested using Model 4 of the Hayes PROCESS macro for SPSS (version 4.2) with 5000 bootstrap samples and 95% bias‐corrected confidence intervals (Hayes 2017); an indirect effect was considered statistically significant when the confidence interval excluded zero. Because all variables were measured concurrently in a cross‐sectional design, the mediation model is interpreted as a statistical decomposition of association and not as evidence of a causal or temporal sequence. Statistical significance was set at *p* < 0.05. Internal consistency of the scales was evaluated using Cronbach's alpha.

### 4.7 Ethical Considerations

The study was conducted in accordance with the Declaration of Helsinki. Ethical approval was obtained from the institutional Research Ethics Committee of the corresponding author's faculty (Decision No: 679.49.04). All participants were at least 18 years of age, so no parental or guardian consent was required. Participants were informed about the aim, scope and voluntary nature of the study, and digital informed consent was obtained prior to data collection. To ensure anonymity and confidentiality, data were stored in password‐protected files accessible only to the research team.

## 5 Results

### 5.1 Subjects (Sample Characteristics)

Participants' mean age was 20.68 ± 2.55 years (range 18–38 years). The majority of students were in their second year (32.5%) or first year (27.5%); 39.3% reported choosing midwifery mainly because of job opportunities and career prospects. Just over half of respondents (54.2%) were satisfied with their profession; 64.7% wished to work in public hospitals; and 90.5% reported a sense of professional identity. Approximately two‐thirds (63.7%) were optimistic about the future of the profession. During clinical practice, 30.5% reported having experienced disgust, 27.5% reported having had negative thoughts about the profession, 9.5% reported avoidance of care and 12.9% reported doubts about their professional competence. The full sample characteristics are presented in Table 1.

### 5.2 Levels of Disgust Sensitivity, Professional Perception and Professional Belonging

Mean total scores were 40.64 ± 10.65 for the DPSS‐R (50.8% of the maximum), 70.96 ± 8.95 for the MVPS (88.7%) and 85.19 ± 12.80 for the MBS (77.4%); using the descriptive criteria defined above, this corresponds to a moderate level of disgust sensitivity and high levels of professional perception and belonging. Cronbach's alpha values ranged from 0.83 to 0.96 across the three total scales, indicating good to excellent internal consistency, and skewness and kurtosis values were within ±1.5, supporting the use of parametric tests. To improve clarity and because only total scores were used in the regression and mediation analyses, the descriptive table reports the three total scale scores; subdimension descriptives are provided in the open dataset (see Data Availability Statement). Descriptive statistics are presented in Table 2.

**TABLE 2:** Descriptive statistics of the total scale scores (*N* = 295).

Scale	Min	Max	Mean ± SD	Skewness/Kurtosis	*α*
DPSS‐R total	16	71	40.64 ± 10.65	0.02/−0.16	0.91
MVPS total	45	80	70.96 ± 8.95	−0.81/−0.14	0.96
MBS total	54	110	85.19 ± 12.80	0.02/−0.43	0.93

*Note:* *α* denotes the Cronbach's alpha internal‐consistency reliability coefficient for the total scale.

Abbreviations: DPSS‐R, Disgust Propensity and Sensitivity Scale–Revised; Max, maximum; MBS, Midwifery Belonging Scale; Min, minimum; MVPS, Midwifery Vocational Perception Scale; SD, standard deviation.

### 5.3 Differences by Sociodemographic and Professional Characteristics

As pre‐specified, these subgroup comparisons were exploratory and were restricted to variables in which all subgroups were adequately sized (Section 4.6). The specific tests are described in Section 4.6; in brief, two‐group comparisons used independent‐samples *t*‐tests and comparisons of adequately sized three‐group variables used one‐way ANOVA (or Welch's test when variances were unequal), followed by Bonferroni‐corrected or Games–Howell post hoc tests. Given the number of comparisons, emphasis is placed on associations significant at *p* < 0.001. Results are summarised in Table 3.

**TABLE 3:** Comparison of mean DPSS‐R, MVPS and MBS scores by sociodemographic and professional characteristics (*N* = 295).

Variable	*n*	DPSS‐R Mean ± SD	MVPS Mean ± SD	MBS Mean ± SD
Year of study				
1st year	81	39.49 ± 11.44	73.10 ± 7.43	88.22 ± 13.38
2nd year	96	41.31 ± 11.49	68.15 ± 9.64	83.85 ± 12.27
3rd year	62	39.53 ± 8.93	73.42 ± 7.08	87.13 ± 11.61
4th year	56	42.38 ± 9.62	69.95 ± 10.18	80.93 ± 12.89
Test/*p*		*F* = 1.161; 0.325	*W* = 7.005; < 0.001	*F* = 4.568; 0.004
Professional satisfaction				
Yes	160	38.02 ± 10.94	73.67 ± 7.39	91.66 ± 10.89
No	30	49.43 ± 8.77	62.63 ± 10.21	69.03 ± 8.60
Undecided	105	42.12 ± 9.02	69.19 ± 8.92	79.93 ± 9.66
Test/*p*		*W* = 20.385; < 0.001	*W* = 21.520; < 0.001	*F* = 82.850; < 0.001
Perceived professional identity				
Yes	267	39.94 ± 10.28	71.87 ± 8.30	86.63 ± 12.31
No	28	47.29 ± 11.96	62.29 ± 10.36	71.39 ± 8.56
Test/*p*		*t* = −3.538; < 0.001	*t* = 5.666; < 0.001	*t* = 6.387; < 0.001
Experience of disgust during practice				
Yes	90	45.63 ± 9.92	68.38 ± 9.61	79.30 ± 12.22
No	205	38.45 ± 10.23	72.09 ± 8.42	87.77 ± 12.20
Test/*p*		*t* = 5.604; < 0.001	*t* = −3.334; 0.001	*t* = −5.487; < 0.001
Negative thoughts				
Yes	81	47.15 ± 8.73	66.48 ± 10.79	77.17 ± 12.20
No	214	38.18 ± 10.28	72.65 ± 7.51	88.22 ± 11.69
Test/*p*		*t* = 7.489; < 0.001	*t* = −4.731; < 0.001	*t* = −7.159; < 0.001
Avoidance of care				
Yes	28	48.96 ± 8.86	65.07 ± 9.38	74.21 ± 10.02
No	267	39.77 ± 10.46	71.57 ± 8.69	86.34 ± 12.52
Test/*p*		*t* = 4.486; < 0.001	*t* = −3.737; < 0.001	*t* = −4.956; < 0.001
Doubt about professional competence				
Yes	38	48.97 ± 9.47	66.08 ± 9.70	74.08 ± 8.74
No	257	39.41 ± 10.27	71.68 ± 8.62	86.83 ± 12.49
Test/*p*		*t* = 5.410; < 0.001	*t* = −3.674; < 0.001	*t* = −6.071; < 0.001

*Note:* Statistical significance was set at *p* < 0.05; given multiplicity, associations at *p* < 0.001 are emphasised. Post hoc tests (Bonferroni/Games–Howell, *p* < 0.05): year of study—MVPS 1 > 2, 3 > 2, 3 > 4; MBS 1 > 2, 1 > 4, 3 > 4. Professional satisfaction—DPSS‐R dissatisfied>undecided > satisfied; MVPS and MBS satisfied>undecided > dissatisfied. Variables with small subgroups (career preference, reason for choosing the profession, perception of the future of the profession) were excluded from inferential comparison and are shown only in Table 1.

Abbreviations: DPSS‐R, Disgust Propensity and Sensitivity Scale–Revised; *F*, one‐way ANOVA; MBS, Midwifery Belonging Scale; MVPS, Midwifery Vocational Perception Scale; *n*, frequency; SD, standard deviation; *t*, independent‐samples *t*‐test; W, Welch test.

Disgust sensitivity differed significantly across groups defined by professional satisfaction, perceived professional identity and all four clinical‐experience variables (experience of disgust, negative thoughts, avoidance of care and doubt about competence; all *p* < 0.001). Disgust scores were higher among students who were dissatisfied or undecided (post hoc: dissatisfied>undecided > satisfied), among those without a sense of professional identity, and among those reporting disgust experiences, negative thoughts, avoidance of care or self‐doubt.

Professional perception differed significantly by year of study, professional satisfaction, perceived professional identity and the four clinical‐experience variables (all *p* < 0.05). For year of study, post hoc tests indicated that first‐ and third‐year students reported more positive perceptions than second‐year students, and satisfied students scored higher than dissatisfied or undecided students. Students with a sense of professional identity and those without disgust, negative thoughts, care avoidance or self‐doubt also scored higher on the MVPS.

Professional belonging followed a similar pattern, with significant differences by year of study, professional satisfaction, perceived professional identity and the four clinical‐experience variables (all *p* < 0.05). Post hoc comparisons showed that first‐ and third‐year students scored higher than fourth‐year students, and that satisfied students scored higher than dissatisfied or undecided students. In response to peer review, variables with small subgroup cell sizes (career preference, reason for choosing the profession and perception of the future of the profession) were not analysed inferentially and appear only as descriptive characteristics in Table 1.

### 5.4 Correlations Among the Scales

Pearson correlation analysis was conducted to examine the bivariate associations among the three total scale scores before modelling. As shown in Table 4, a statistically significant, strong, positive correlation was identified between the MVPS total score and the MBS total score (*r* = 0.64, *p* < 0.001). The DPSS‐R total score showed a significant weak negative correlation with the MVPS total score (*r* = −0.21, *p* < 0.001) and a significant moderate negative correlation with the MBS total score (*r* = −0.34, *p* < 0.001).

**TABLE 4:** Correlations among the total scale scores.

Variable	1	2	3
1. DPSS‐R total	—		
2. MVPS total	−0.21**	—	
3. MBS total	−0.34**	0.64**	—

Abbreviations: DPSS‐R, Disgust Propensity and Sensitivity Scale–Revised; MBS, Midwifery Belonging Scale; MVPS, Midwifery Vocational Perception Scale; *r*, Pearson correlation coefficient.

** *p* < 0.01.

### 5.5 Hierarchical Regression Analysis

Hierarchical regression analysis was conducted to examine the predictive contribution of professional perception and disgust sensitivity to professional belonging (Table 5). Tolerance and VIF values (tolerance = 0.955; VIF = 1.048) and the Durbin–Watson statistic (2.047) indicated no concerns regarding multicollinearity or autocorrelation.

**TABLE 5:** Hierarchical regression analysis predicting professional belonging.

Model	Independent variable	*B*	SE(B)	*β*	*t*	*p*	95% CI [LB, UB]
1	Constant	20.385	4.597	—	4.434	< 0.001	[11.337, 29.433]
	MVPS total	0.913	0.064	0.639	14.207	< 0.001	[0.787, 1.040]
2	Constant	35.234	5.430	—	6.489	< 0.001	[24.548, 45.920]
	MVPS total	0.849	0.064	0.594	13.370	< 0.001	[0.724, 0.974]
	DPSS‐R total	−0.253	0.053	−0.211	−4.746	< 0.001	[−0.358, −0.148]

*Note:* Model 1: *R* ^2^ = 0.408, Adj. *R* ^2^ = 0.406, *F*(1, 293) = 201.829, *p* < 0.001. Model 2: *R* ^2^ = 0.450, Adj. *R* ^2^ = 0.447, Δ*R* ^2^ = 0.042, Δ*F*(1, 292) = 22.520, *p* < 0.001; tolerance = 0.955; VIF = 1.048; Durbin–Watson = 2.047.

Abbreviations: *B*, unstandardised regression coefficient; CI, confidence interval; DPSS‐R, Disgust Propensity and Sensitivity Scale–Revised; LB/UB, lower/upper bound; MVPS, Midwifery Vocational Perception Scale; SE(B), standard error of B; *β*, standardised regression coefficient.

In Step 1, MVPS total score alone explained 40.8% of the variance in MBS (*R* ^2^ = 0.408, *F*(1, 293) = 201.829, *p* < 0.001), with a strong positive standardised coefficient (*β* = 0.639, *t* = 14.207, *p* < 0.001). In Step 2, the addition of DPSS‐R increased the explained variance to 45.0% (*R* ^2^ = 0.450, Δ*R* ^2^ = 0.042, Δ*F*(1, 292) = 22.520, *p* < 0.001). MVPS retained its positive coefficient (*β* = 0.594, *t* = 13.370, *p* < 0.001), while DPSS‐R showed a significant negative coefficient (*β* = −0.211, *t* = −4.746, *p* < 0.001). Thus, professional perception was the strongest predictor of belonging, while disgust sensitivity made a small but statistically significant additional negative contribution of about 4% of the variance.

### 5.6 Mediation Analysis

**FIGURE 1:**
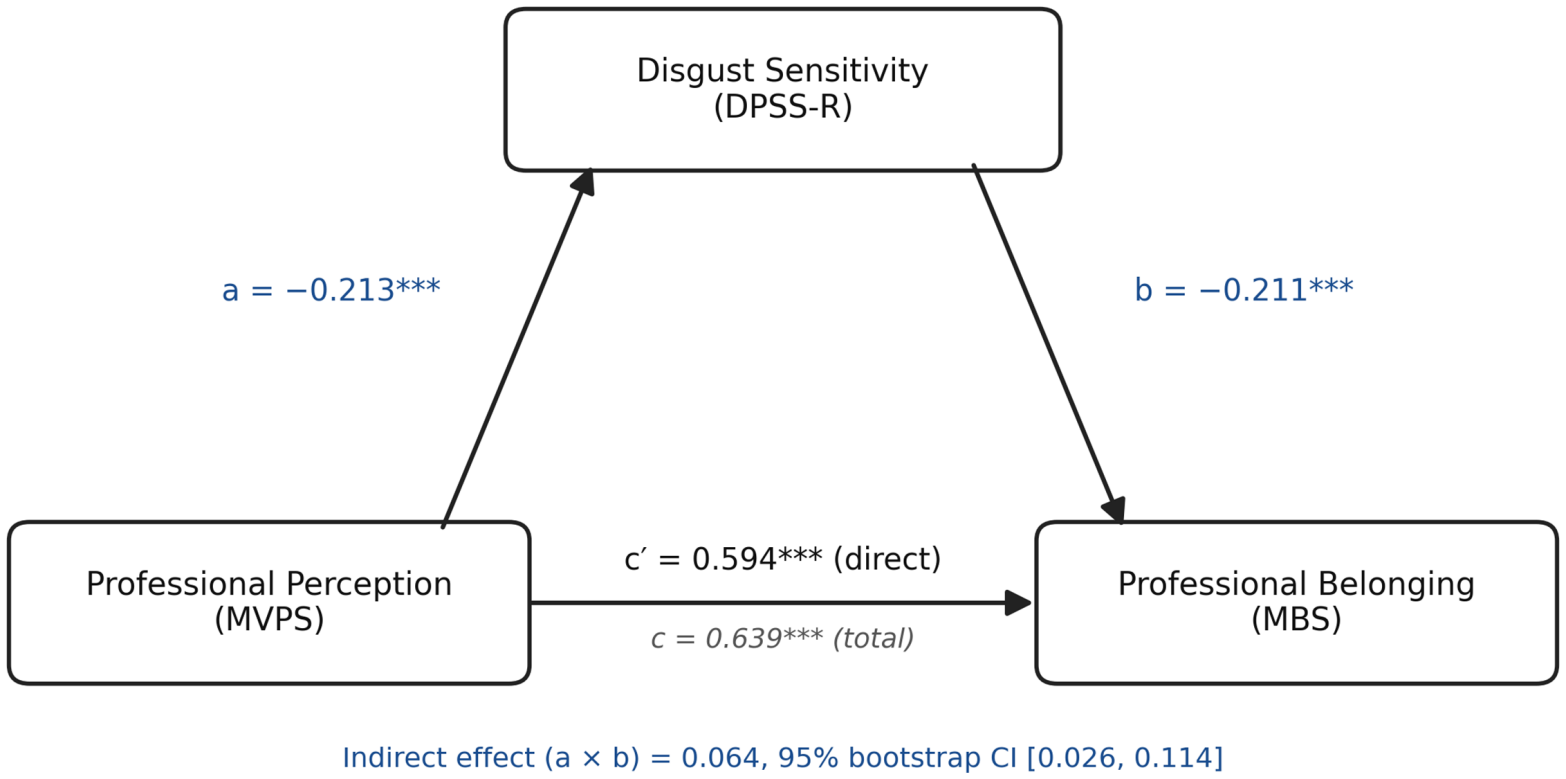
Statistical mediation model of the association between professional perception (MVPS) and professional belonging (MBS) through disgust sensitivity (DPSS‐R). Standardised coefficients (*β*) are shown; ***p < 0.001. The model is cross‐sectional and does not establish causal direction.

To examine whether disgust sensitivity statistically mediates the association between professional perception and professional belonging, Model 4 of the Hayes PROCESS macro was used with 5000 bootstrap samples (Table 6; Figure 1). MVPS was negatively associated with DPSS‐R (a path: *β* = −0.213, *B* = −0.254, SE = 0.068, *p* < 0.001). DPSS‐R was negatively associated with MBS, controlling for MVPS (b path: *β* = −0.211, *B* = −0.253, SE = 0.053, *p* < 0.001). The total association of MVPS with MBS (c path: *β* = 0.639, *B* = 0.913, *p* < 0.001) was reduced but remained significant after the mediator was added (c′ path: *β* = 0.594, *B* = 0.849, *p* < 0.001). The bootstrap‐derived indirect effect was statistically significant, as its 95% bias‐corrected confidence interval excluded zero (a × b: *β* = 0.045, indirect effect = 0.064, BootSE = 0.023, 95% CI [0.026, 0.114]), indicating partial statistical mediation. The full model accounted for 45.0% of the variance in MBS (*R* ^2^ = 0.450, *F*(2, 292) = 119.587, *p* < 0.001). Because all variables were measured at the same time, this pattern is consistent with—but does not establish—the proposed perception → disgust → belonging sequence; alternative orderings cannot be ruled out.

**TABLE 6:** Mediating role of disgust sensitivity in the relationship between professional perception and professional belonging.

Path	Effect (B)	SE	*β*	95% CI [LB, UB]	*p*
a path: MVPS → DPSS‐R	−0.254	0.068	−0.213	—	< 0.001
b path: DPSS‐R → MBS (controlling MVPS)	−0.253	0.053	−0.211	—	< 0.001
c path (total): MVPS → MBS	0.913	0.064	0.639	[0.787, 1.040]	< 0.001
c′ path (direct): MVPS → MBS	0.849	0.064	0.594	[0.724, 0.974]	< 0.001
Indirect effect (a × b): MVPS → DPSS‐R → MBS	0.064	0.023	0.045	[0.026, 0.114]	—

*Note:* Mediation tested using Hayes PROCESS Model 4 with 5000 bootstrap samples. The indirect effect is considered statistically significant because its 95% bias‐corrected bootstrap confidence interval (BootSE = 0.023) excludes zero; direct and total effects are significant at *p* < 0.001. Mediator model: *R* ^2^ = 0.045, *F*(1, 293) = 13.935, *p* < 0.001. Outcome model: *R* ^2^ = 0.450, *F*(2, 292) = 119.587, *p* < 0.001.

Abbreviations: DPSS‐R, Disgust Propensity and Sensitivity Scale–Revised; MBS, Midwifery Belonging Scale; MVPS, Midwifery Vocational Perception Scale.

## 6 Discussion

This descriptive, cross‐sectional and correlational study examined the levels and interrelationships of disgust sensitivity, professional perception and professional belonging in midwifery students, and tested whether disgust sensitivity statistically mediates the relationship between professional perception and belonging. Three principal findings emerged. First, students reported high levels of professional perception and belonging together with a moderate level of disgust sensitivity. Second, professional perception was the strongest correlate of professional belonging, while disgust sensitivity made a small, independent and negative additional contribution. Third, disgust sensitivity partially and statistically mediated the association between professional perception and professional belonging. Taken together, and within the limits of a cross‐sectional design, these findings suggest that both professional perception and emotional responses such as disgust are associated with professional belonging; they do not establish that one causes the other.

### 6.1 Levels of Professional Perception, Belonging and Disgust Sensitivity

The high levels of professional perception (MVPS 70.96/80) and professional belonging (MBS 85.19/110) observed in our sample are consistent with previous Turkish studies of midwifery students (Demir and Taşpınar 2021; Yıldırım et al. 2022; Yılmaz and Siyahtaş 2024) and with international qualitative work that has highlighted strong identification with woman‐centred care among midwifery students (Alghamdi and Alobaid 2026; Sun et al. 2022). The strong positive correlation we observed between perception and belonging (*r* = 0.64) is in line with the view that internalising the profession's values and roles—through socialisation, role modelling and clinical participation—is associated with stronger attachment to the professional community (Mbalinda et al. 2024; Stephenson and Cox 2025; Thomas et al. 2023).

The moderate level of disgust sensitivity (DPSS‐R 40.64/80) is broadly consistent with the literature, although direct comparisons should be made cautiously because of methodological differences and because the DPSS‐R captures both disgust propensity and disgust sensitivity, which are related but distinct constructs (Van Overveld et al. 2006). In nursing students, Özkan et al. (2021) and Adagide et al. (2024) reported elevated disgust sensitivity using related instruments. Our somewhat lower scores may reflect the specific characteristics of midwifery practice—where exposure to bodily products is normalised through the centrality of childbirth—and the predominantly female sample in midwifery education. Importantly, almost one third (30.5%) of students explicitly reported having experienced disgust during clinical practice, while smaller but clinically meaningful proportions reported negative thoughts (27.5%), avoidance of care (9.5%) and doubts about competence (12.9%). These findings mirror phenomenological work showing that nursing and midwifery students often experience disgust but rarely verbalise it (Bandadi et al. 2024; Cho et al. 2023; Hadjittofi et al. 2022).

### 6.2 Disgust Sensitivity, Professional Perception and Professional Belonging

Disgust sensitivity was negatively associated with professional perception (*r* = −0.21) and more strongly with professional belonging (*r* = −0.34). From a theoretical perspective, it is plausible that professional belonging shows a stronger association: belonging is an inherently affective construct grounded in feeling welcomed, valued and connected to a profession (Folkvord and Risa 2023), and repeated negative emotional experiences may be related to a weaker connection. According to the Behavioural Immune System perspective (Van Overveld et al. 2006), disgust functions as a frontline defence motivating avoidance of pathogen cues. When disgust is experienced repeatedly, particularly in a caregiving role, this avoidance tendency may be misaligned with the professional norms of approach, contact and intimate care, and may be associated with a reduced sense of fit between self and profession (Hadjittofi et al. 2020; Salmani Mood and Nasiri 2025; Troisi et al. 2022).

The hierarchical regression analysis showed that professional perception was the strongest predictor of professional belonging, while disgust sensitivity made a small, independent and negative contribution, increasing the explained variance from 40.8% to 45.0%. Although this additional 4.2% is modest, it was statistically robust and is consistent with quantitative evidence that higher disgust sensitivity is associated with reduced caring behaviours among nursing students and clinical nurses (Doğan et al. 2025; Özkan et al. 2021; Song et al. 2024) and with lower willingness to care for older adults (Adagide et al. 2024). Song et al. (2024) reported that disgust sensitivity weakened the association between proactive personality and caring behaviour via emotional intelligence in a moderated mediation model among clinical nurses, suggesting that disgust may act as an emotional brake on the positive contribution of personality. Our findings extend this emerging picture to midwifery students by showing that disgust sensitivity is also associated with belonging, the affective core of professional identity, while underscoring that perception remains the dominant correlate.

### 6.3 The Mediating Role of Disgust Sensitivity

To our knowledge, this study provides the first quantitative evidence in midwifery education that disgust sensitivity may partially and statistically mediate the association between professional perception and professional belonging. The indirect pathway is consistent with the possibility that students with more positive professional perception experience less disgust during clinical practice, which is in turn associated with stronger belonging. Two complementary mechanisms may underlie this association. Cognitively, students who view midwifery as meaningful and valuable may be more likely to reframe potentially aversive experiences (e.g., body fluids, intimate examinations, perinatal loss) as integral and purposeful aspects of care, dampening the felt intensity of disgust (Cho et al. 2023; Hadjittofi et al. 2022). Behaviourally, a strong professional schema may direct attention towards woman‐centred goals rather than pathogen cues, reducing Behavioural Immune System activation in line with appraisal‐based accounts of the system (Troisi et al. 2022).

Crucially, the design of this study is cross‐sectional, and all three variables were measured at the same time. Statistical mediation under these conditions does not establish the temporal or causal ordering implied by the model; the data are equally compatible with alternative sequences (e.g., belonging shaping perception, or a third variable accounting for both), and the indirect effect should be read as a decomposition of association rather than as evidence of a causal mechanism. The mediation was also partial: the direct association between professional perception and belonging remained large after accounting for disgust, indicating that perception relates to belonging largely through pathways other than disgust. These observations complement qualitative work suggesting that disgust experiences may threaten professional identity construction unless they are processed into the self‐narrative of becoming a competent caregiver (Warmington et al. 2022), and they point to the need for longitudinal and experimental designs to test directionality.

### 6.4 Sociodemographic and Clinical Correlates

In these analyses, patterns of difference across the retained sociodemographic and clinical variables were broadly consistent with the main findings. Students who were satisfied with their profession and who held a clear sense of professional identity reported lower disgust sensitivity and higher perception and belonging. Conversely, students reporting disgust during practice, negative thoughts, care avoidance or doubts about competence reported lower perception and belonging. These patterns are consistent with the literature linking positive professional attitudes and a clear professional identity with stronger professional commitment (Çingöl et al. 2020), and with qualitative work documenting how high‐intensity working conditions, feeling unsupported and emotional events such as perinatal death may impede professional identity development among midwifery students (Shen et al. 2022; Sun et al. 2022).

Year‐of‐study differences were also informative. First‐ and third‐year students reported more positive professional perception and belonging than fourth‐year students, while disgust sensitivity did not vary significantly across year groups. This pattern is unlikely to reflect simple desensitisation through exposure; rather, it may be related to the cumulative influence of the clinical learning environment. Negative socialisation experiences—such as bullying on placement, lack of supervisory support and exposure to suboptimal role models—have been associated with a reduced sense of belonging and erosion of professional values among midwifery students (Capper et al. 2021; Javornická et al. 2024).

### 6.5 Implications for Nursing and Midwifery Practice

These findings translate into several concrete, evidence‐based applications for nursing and midwifery education and clinical practice, while recognising that the modest effect of disgust sensitivity means it should be addressed as one component of a broader strategy to support professional belonging rather than a primary lever. First, because disgust appears common yet rarely discussed, nurse and midwife educators and clinical mentors can legitimise it within routine clinical supervision and debriefing—explicitly naming disgust as a normal, manageable response—so that students are less likely to conceal it or to disengage from care (Hadjittofi et al. 2020; Salmani Mood and Nasiri 2025). Second, graduated and supervised clinical exposure, supported by high‐fidelity or virtual‐reality simulation before students encounter high‐contact or distressing procedures, can build confidence and reduce anxiety while protecting women's safety and dignity (Moloney et al. 2022; Yahya et al. 2024). Third, structured reflective debriefing after emotionally demanding events, such as perinatal loss, can help students articulate, normalise and reframe disgust as part of becoming a competent caregiver. Fourth, supportive mentorship and positive role modelling, which buffer negative emotions and strengthen professional identity, should be embedded throughout the programme rather than only in early placements (Arundell et al. 2024; Folkvord and Risa 2023). Finally, because students who are dissatisfied or who doubt their competence also report higher disgust and weaker belonging, programmes could incorporate brief self‐monitoring of distress and signpost accessible psychological support, so that students whose belonging is most at risk can be identified and supported early. Together, these measures give nurse educators specific, feasible ways to translate the present findings into curriculum and placement design.

### 6.6 Strengths and Limitations of the Work

This study has several strengths, including a relatively large sample of midwifery students (*N* = 295), a high response rate (83.8%), use of validated psychometric instruments with good internal consistency, and the application of bootstrap mediation analysis to test a theoretically grounded mechanism. Reporting followed the STROBE guideline for cross‐sectional studies, and the de‐identified dataset and analysis syntax have been made openly available (see Data Availability Statement). Several limitations should nonetheless be considered. First, because of the cross‐sectional design, causal and temporal inferences are not possible; the directionality of the perception–disgust–belonging pathway must be established in longitudinal and experimental studies. Second, all data were collected through a single self‐administered online questionnaire, so the findings may be affected by social desirability and common‐method bias; we did not conduct a formal test (e.g., Harman's single‐factor test) of common‐method variance, and future studies should incorporate procedural and statistical remedies and, where possible, multi‐source data. Third, the survey package was relatively long and no attention‐check or instructed‐response items were included, so inattentive responding cannot be excluded—a recognised limitation of online survey research. Fourth, the regression and mediation models did not adjust for the sociodemographic and professional variables that were significant in the bivariate analyses; covariate‐adjusted models are an important next step. Fifth, in response to peer review, subgroup comparisons involving variables with small cell sizes (career preference, reason for choosing the profession and perception of the future of the profession) were removed from the analyses; the group comparisons that remain all involved adequately sized subgroups but, given their number, were treated as exploratory with emphasis on associations significant at *p* < 0.001. Sixth, the DPSS‐R captures both disgust propensity and disgust sensitivity, and the extent to which findings are specific to sensitivity rather than propensity should be examined with dedicated measures. Finally, the study was conducted at a single state university in southeastern Türkiye with a predominantly female cohort, which may restrict generalisability to other regions, programmes, cultural contexts and to male students; several potentially relevant factors (personality, prior healthcare or caregiving experience, religiosity, placement type and duration, supervisory support) were not measured.

### 6.7 Recommendations for Further Research

Longitudinal studies are needed to clarify the temporal dynamics of disgust, professional perception and professional belonging across the trajectory of midwifery education and into early professional practice. Experimental and quasi‐experimental designs should test how educational interventions (e.g., high‐fidelity simulation, virtual reality, structured reflective debriefing and mentorship programmes) influence disgust regulation and professional belonging, as these designs—unlike the present cross‐sectional study—can support causal inference. Future work should also use covariate‐adjusted models, report formal assessments of common‐method bias, employ attention checks and incorporate additional individual (personality, prior caregiving experience, religiosity) and contextual (placement quality, supervisory support, organisational culture) predictors. Cross‐cultural and multi‐site studies, including male midwifery students where applicable, and the continued adoption of open‐science practices such as data and code sharing, would further strengthen the robustness and generalisability of this line of research.

## 7 Conclusion

Midwifery students in this study reported high professional perception and belonging together with a moderate level of disgust sensitivity. Professional perception was the strongest correlate of professional belonging, and disgust sensitivity showed a small, independent negative association that partially and statistically mediated the perception–belonging relationship. Within the limits of a cross‐sectional design, these findings indicate that professional perception and emotional responses such as disgust are both associated with professional belonging, without implying a causal sequence. Curricula that combine technical training with structured emotional support—through graduated clinical exposure, simulation‐based learning, reflective practice and supportive mentorship—may help strengthen students' sense of belonging and, in turn, support the quality of midwifery care; this possibility should be tested directly in future interventional research.

## Author Contributions

**Ayşe Elkoca:** investigation, writing – original draft, conceptualization, writing – review and editing. **Songül Kekil:** investigation, writing – original draft, writing – review and editing. **Betül Kaplan:** conceptualization, methodology, formal analysis, data curation, writing – original draft, writing – review and editing, supervision.

## Funding

The authors have nothing to report.

## Disclosure

*Statistical declaration*: The statistical analyses reported in this manuscript were designed and performed by [Betül KAPLAN], who has expertise in quantitative research methods, and were independently reviewed for appropriateness by the co‐authors. All analyses were conducted in IBM SPSS Statistics version 30 (IBM Corp., Armonk, NY, USA) with the Hayes PROCESS macro version 4.2. The complete analysis syntax and the de‐identified dataset are openly available in the repository (see Data Availability Statement), enabling independent verification of all reported results.

## Ethics Statement

This study was approved by the Gaziantep Islam Science and Technology University Non‐Interventional Clinical Research Ethics Committee. Approval date: 17.09.2025. Approval/decision number: 679.49.04. The study was conducted in accordance with the Declaration of Helsinki, and written/digital informed consent was obtained from all participants, all of whom were aged 18 years or older.

## Conflicts of Interest

The authors declare no conflicts of interest.

## Supporting information


**File S1:** STROBE statement—Checklist for cross‐sectional studies.

## Data Availability

The de‐identified dataset and analysis syntax supporting the findings are openly available in the Open Science Framework (OSF) at https://osf.io/my‐projects?tab=1&page=1 (DOI: https://doi.org/10.17605/OSF.IO/B47MX). A view‐only anonymized link for peer review: https://osf.io/b47mx/overview.
