# Supplementary material for: The Mediating Role of Disgust Sensitivity in the Relationship Between Professional Perception and Professional Belonging Among Midwifery Students: A Cross‐Sectional Study
Source: Nurs Open. 2026 Aug 16;13(8):e70749. doi: 10.1002/nop2.70749 (PMC13478568; doi:10.1002/nop2.70749)
Supplement: Supplementary file 1 [file NOP2-13-e70749-s001.docx]

**Supplementary File S1. STROBE Statement—Checklist for Cross-Sectional Studies**

Reporting locations refer to sections of the revised manuscript. STROBE = Strengthening the Reporting of Observational Studies in Epidemiology.

| **Section / Item** | **No.** | **STROBE recommendation** | **Reported in** |
| --- | --- | --- | --- |
| Title and abstract | 1 | (a) Indicate the study design in the title or abstract. (b) Provide an informative and balanced summary in the abstract. | Title; Abstract |
| **Introduction** | | | |
| Background/rationale | 2 | Explain the scientific background and rationale. | Section 1; Section 2 |
| Objectives | 3 | State specific objectives, including any pre-specified hypotheses. | Section 3.1 |
| **Methods** | | | |
| Study design | 4 | Present key elements of study design early in the paper. | Section 4.1; Abstract (Design) |
| Setting | 5 | Describe setting, locations and relevant dates, including recruitment and data collection periods. | Sections 4.2, 4.4 |
| Participants | 6 | Give eligibility criteria and sources/methods of selection of participants. | Sections 4.2, 4.3 |
| Variables | 7 | Clearly define all outcomes, exposures, predictors and potential confounders. | Sections 4.5, 4.6 |
| Data sources/measurement | 8 | Give sources of data and details of methods of assessment for each variable of interest. | Sections 4.4, 4.5 |
| Bias | 9 | Describe any efforts to address potential sources of bias. | Sections 4.6, 6.6 |
| Study size | 10 | Explain how the study size was arrived at. | Section 4.3 |
| Quantitative variables | 11 | Explain how quantitative variables were handled in the analyses. | Section 4.6 |
| Statistical methods | 12 | Describe all statistical methods, control for confounding, subgroups/interactions, missing data, and sensitivity analyses. | Section 4.6 |
| **Results** | | | |
| Participants | 13 | Report numbers at each stage, reasons for non-participation; consider a flow diagram. | Section 4.3; Section 5.1 |
| Descriptive data | 14 | Give characteristics of study participants and information on exposures and potential confounders. | Section 5.1; Table 1 |
| Outcome data | 15 | Report numbers of outcome events or summary measures. | Sections 5.2–5.6; Tables 2–6 |
| Main results | 16 | Give unadjusted and, where relevant, adjusted estimates and their precision (e.g., 95% CI). | Sections 5.4–5.6; Tables 4–6; Figure 1 |
| Other analyses | 17 | Report other analyses done (subgroups, interactions, sensitivity). | Section 5.3; Table 3 |
| **Discussion** | | | |
| Key results | 18 | Summarise key results with reference to study objectives. | Section 6 (opening); 6.1–6.4 |
| Limitations | 19 | Discuss limitations, sources of potential bias or imprecision, and direction/magnitude of bias. | Section 6.6 |
| Interpretation | 20 | Give a cautious overall interpretation considering objectives, limitations, multiplicity, and other evidence. | Sections 6.1–6.5, 6.7 |
| Generalisability | 21 | Discuss the generalisability (external validity) of the study results. | Section 6.6 |
| **Other information** | | | |
| Funding | 22 | Give the source of funding and the role of funders. | Back matter (Funding) |

Note. An Explanation and Elaboration article discusses each checklist item and is available with the STROBE Statement at https://www.strobe-statement.org.
